# Higher perceived HRQoL in Moroccan children with asthma and their parents

**DOI:** 10.11604/pamj.2015.21.18.6191

**Published:** 2015-05-08

**Authors:** Monique Theodora Maria Veenstra-van Schie, Kelly Coenen, Hendrik Maria Koopman, Florens Gerard Adriaan Versteegh

**Affiliations:** 1 Groene Hart Ziekenhuis, Department of Pediatrics, Gouda, the Netherlands; 2 Leiden University, Department of Clinical Psychology, Leiden, the Netherlands; 3 Ghent University Hospital, Department of Pediatrics, Ghent, Belgium

**Keywords:** Asthma, cultural differences, children, parents

## Abstract

We explored the differences in the perceived HRQoL between children with asthma from Moroccan and Dutch descent and their parents. In total 33 children (aged 6-18 years) from Moroccan (16) and Dutch descent (17) and their parents participated. All children were currently under treatment in a general hospital in the Netherlands. Generic and asthma specific HRQoL were assessed (DUX-25, DISABKIDS, PAQLQ). Significant differences were found on the DUX-25 subscales physical, emotional and home functioning. Children and parents from Dutch descent reported a lower HRQoL. The findings of this study are contrary with previous research. Results can be explained by the individualistic-collectivistic dimension, socially desirability, language and the feeling of miscomprehension. If this explanation makes sense health care workers have to invest in a good relationship with especially immigrant children and their parents, so they will have enough confidence to talk more openly about their physical as well as their psycho-social complaints.

## Introduction

The Netherlands is a multi-cultural society with 17 million residents of which approximately 20% is from nonnative descendence. The three largest ethnic groups are immigrants from Suriname (2,3%), Turkey (2,3%) and Morocco (2,1%) [1]. In the Gouda region in the central part of the Netherlands the total percentage of children from Moroccan descent aged 6-18 years is 8% [2]. In the Groene Hart Ziekenhuis (GHZ), a general teaching hospital, a group of about 70 children with asthma, aged 6-18 years from Moroccan descent are treated. Several determinants were found to be of influence on the health-related quality of life (HRQoL), with ethnicity being one of them [1, 3]. None of these studies explored the Moroccan population. Therefore we investigated the differences in the perceived HRQoL between children with asthma and their parents from Moroccan (Berber and Arab) and Dutch descent.

## Methods

Children diagnosed with asthma and currently under treatment in the GHZ were tested, using generic and asthma specific HRQoL instruments (DUX-25 [4], DISABKIDS [5] and the PAQLQ [6]. Parents filled in proxy versions of the questionnaires. Data were analyzed using SPSS (version 18).

## Results

In this study 33 children were included (64% male), 16 from Moroccan and 17 matched children and parents from Dutch descent. The age of all children ranged from 6-16 years and of the parents from 31-56 years. Generally, mothers completed the questionnaires (85%). No significant differences (age, education level, and asthma severity) were found between the Moroccan and Dutch groups. Independent t-tests showed that the two parent groups only differed significantly on the DUX-25 overall score (p = 0.024) and the DUX-25 subscale physical (p = 0.004). (table 1, upper part). Moroccan parents showed higher overall and physical subscale scores indicating a better HRQoL. Moroccan children differed significantly on the DUX-25 overall score (p = 0.002) and the subscales physical (p = 0.006)) (table 1, lower part), emotional (p =0.003) and home functioning (p = 0.006) also indicating a better HRQoL compared to children from Dutch descent. No significant differences were seen on the DISABKIDS and the PAQLQ questionnaires.

**Table 1:** Unadjusted mean scores#, the range and standard deviations (SD) of the DUX-25, DISABKIDS and PAQLQ in parents of children with asthma from Dutch and Moroccan descent (upper part) and in children with asthma from Dutch and Moroccan descent (lower part)

HRQoL instruments and subscales *(N=33)*	Dutch (n=17) Mean ± SD	Dutch Mean range	Moroccan (n=16) Mean ± SD	Moroccan Mean range	*p^x^*
**Parents**					
**DUX-25**					
Overall score	1.89 ± 0.51	1.07 -2.81	1.47 ± 0.51	1.00 -2.56	**0.024**
Physical	2.10 ± 0.57	1.00 -3.00	1.50 ± 0.55	1.00 -3.00	**0.004**
Emotional	1.96 ± 0.54	1.00 -2.83	1.63 ± 0.63	1.00 -2.83	0.110
Social	1.84 ± 0.62	1.00 -3.00	1.49 ± 0.50	1.00 -2.57	0.084
Home Functioning	1.62 ± 0.51	1.00 -2.60	1.31 ± 0.48	1.00 -2.40	0.083
**DISABKIDS**					
Overall score	1.88 ± 0.40	1.35 -2.70	1.95 ± 0.56	1.17 -3.00	0.680
Life	1.86 ± 0.57	1.10 -3.10	1.82 ± 0.62	1.10 -3.20	0.849
Medication	2.85 ± 1.17	1.00 -5.00	2.56 ± 0.93	1.50 -5.00	0.434
Worry	1.84 ± 0.51	1.20 -3.20	2.13 ± 0.77	1.00 -3.60	0.217
Impact	1.98 ± 0.54	1.00 -3.00	2.15 ± 0.85	1.00 -3.17	0.507
**PAQLQ**					
Overall score	2.05 ± 0.83	1.00 -3.52	2.07 ± 0.90	1.00 -3.74	0.942
Activity limitation	2.66 ± 1.24	1.00 -4.40	2.11 ± 1.34	1.00 -4.80	0.235
Symptoms	2.12 ± 0.91	1.00 -4.00	2.21 ± 1.07	1.00 -4.50	0.800
Emotional function	1.48 ± 0.67	1.00 -3.00	1.87 ± 0.94	1.00 -3.88	0.185
**Children**					
**DUX-25**					
Overall score	1.87 ± 0.37	1.37 -2.56	1.40 ± 0.29	1.04 -1.81	**0.002**
Physical	2.07 ± 0.55	1.00 -3.00	1.41 ± 0.31	1.00 -2.00	**0.006**
Emotional	2.07 ± 0.36	1.00 -2.83	1.52 ± 0.42	1.00 -2.17	**0.052**
Social	1.71 ± 0.46	1.00 -2.57	1.41 ± 0.31	1.00 -1.86	0.121
Home Functioning	1.61 ± 0.42	1.00 -2.40	1.18 ± 0.31	1.00 -2.00	**0.006**
**DISABKIDS**					
Overall score	1.85 ± 0.46	1.04 -2.61	1.80 ± 0.49	1.17 -3.00	0.715
Life	1.74 ± 0.51	1.00 -2.60	1.72 ± 0.71	1.00 -3.50	0.688
Medication	2.68 ± 1.31	1.00 -5.00	2.28 ± 0.98	1.00 -5.00	0.413
Worry	1.92 ± 0.61	1.20 -3.20	1.94 ± 0.70	1.00 -3.20	0.637
Impact	2.04 ± 0.69	1.17 -3.67	1.97 ± 0.66	1.00 -3.17	0.641
PAQLQ					
Overall score	2.10 ± 0.68	1.00 -3.48	2.10 ± 0.82	1.09 -4.17	0.815
Activity limitation	2.82 ± 1.27	1.00 -4.40	2.33 ± 1.18	1.00 -4.60	0.272
Symptoms	2.16 ± 0.80	1.00 -4.00	2.08 ± 0.78	1.00 -3.90	0.409
Emotional function	1.49 ± 0.54	1.00 -2.75	1.84 ± 1.07	1.00 -4.38	0.288

x range mean scores: 1 = high HRQoL, 5= low HRQoL. *Significant ***p*value**s in **bold**

## Discussion

In this study no differences were found on the asthma specific HRQoL instruments PAQLQ and DISABKIDS, maybe because these questionnaires are primarily disease based [5–7]. However, compared to other studies where the general HRQoL has shown to be lower in immigrant groups [1, 3], our findings showed the opposite. The significant differences on the DUX-25 (subscales overall, physical, emotional and home functioning) indicated that Dutch children and their parents experience lower HRQoL. A possible explanation could be the cultural influences on the question comprehension and response. People from Moroccan descent have a more collectivistic culture, meaning that they see their self as part of a group instead of independent from a group (more individualistic culture) [8]. More over, it is shown that people in a collectivistic culture are less assertive and direct in their communication [8] and are likely to give more socially desirable responses (no complaints) and don't talk about the negative aspects of having asthma [9]. Small sample size cannot explain the different results on the general HRQoL scores in this study. Despite repeated effort it proved very difficult to include participants from the Moroccan population. However, parents of Moroccan descent became very communicative about their child's asthma while completing the general HRQoL DUX-25 questionnaire. The questions of the DUX-25 are short and clear and invite to talk about everyday activities and to give examples. The DUX-25 answer possibilities (smileys) proved to be more accessible for most people, especially for those of whom the Dutch language is not their native language. Language can be an important determinant in explaining cultural differences in the reported HRQoL [8]. Not only the difficulties of understanding the Dutch language may explain the differences in the perceived HRQoL. Nonnative speakers may not be familiar with the many different terms that can be used to specify disease and complaints in a language, leading to less differentiation in the response. Another explanation for the fact that parents and children of Moroccan descent experienced a higher HRQoL could be that the parents were born in Morocco where there were different expectations regarding health and healthcare [10]. They are raising their children with the same ideas and beliefs about disease as their parents had. Moroccan people are more comfortable in talking about physical complaints, contrary to psycho-social complaints they rather not discuss [8]. A reason may be that they would feel ashamed for their psycho-social complaints or that they would feel miscomprehended by the Western health care system. This feeling of miscomprehension might be the reason of the attenuated answering of the questions.

## Conclusion

The findings of this study are contrary with previous research. Results can be explained by the individualistic-collectivistic dimension, socially desirability, language and the feeling of miscomprehension. If this explanation makes sense health care workers have to invest in a good relationship with especially immigrant children and their parents, so they will have enough confidence to talk more openly about their physical as well as their psycho-social complaints.
